# Enhanced Repaired Enthesis Using Tenogenically Differentiated Adipose-Derived Stem Cells in a Murine Rotator Cuff Injury Model

**DOI:** 10.1155/2022/1309684

**Published:** 2022-05-14

**Authors:** Yang Chen, Yan Xu, Guoyu Dai, Qiang Shi, Chunyue Duan

**Affiliations:** ^1^Department of Spine Surgery and Orthopaedics, Xiangya Hospital, Central South University, Changsha, China 410008; ^2^National Clinical Research Center for Geriatric Disorders, Xiangya Hospital, Central South University, Changsha, China 410008; ^3^Department of Spine Surgery, The Affiliated Changsha Central Hospital, Hengyang Medical School, University of South China, Changsha, China 410004

## Abstract

Rotator cuff tear (RCT) is among the most common shoulder injuries and is prone to rerupture after surgery. Selecting suitable subpopulations of stem cells as a new specific cell type of mesenchymal stem cells has been increasingly used as a potential therapeutic tool in regenerative medicine. In this study, murine adipose-derived SSEA-4+CD90+PDGFRA+ subpopulation cells were successfully sorted, extracted, and identified. These cells showed good proliferation and differentiation potential, especially in the direction of tendon differentiation, as evidenced by qRT-PCR and immunofluorescence. Subsequently, we established a murine rotator cuff injury model and repaired it with subpopulation cells. Our results showed that the subpopulation cells embedded in a fibrin sealant significantly improved the histological score, as well as the biomechanical strength of the repaired tendon enthesis at four weeks after surgery, compared with the other groups. Hence, these findings indicated that the subpopulation of cells could augment the repaired enthesis and lead to better outcomes, thereby reducing the retear rate after rotator cuff repair. Our study provides a potential therapeutic strategy for rotator cuff healing in the future.

## 1. Introduction

Rotator cuff tear (RCT) is a common clinical problem that often leads to shoulder pain and eventually disability, especially in youth sports participants and the elderly [1, 2]. Despite advances in surgical reattachment of the tendon to the bone, the failure rates of rotator cuff repair remain high [3, 4]. Moreover, instead of regenerating the specialized tendon enthesis, which can effectively disperse stress from the muscle, the fibrovascular scar tissue is prone to the formation with poor mechanical reinforcement after surgery [5, 6]. Hence, new repair strategies are required to enhance collagen organization and mechanical strength and prevent the formation of scar tissue.

Mesenchymal stem cells (MSCs) are pluripotent cells with capacities for self-renewal and multiple differentiations [7, 8]. In recent years, stem cell therapies have been proposed as a treatment option for rotator cuff tear by reducing muscle atrophy, fibrosis, and fatty degeneration [9, 10]. It is worth noting that adipose-derived mesenchymal stem cells (ADSCs) and bone marrow mesenchymal stem cells (BMSCs) are the most widely used in the musculoskeletal field [11, 12]. Furthermore, compared with BMSCs, ADSCs are easily harvested from the fat of most animals and humans [13]. However, optimal regeneration has not been achieved with these transplanted MSCs. Therefore, there is an urgent need to find a new subpopulation of stem progenitor cells to influence the regeneration of injured tendon enthesis.

Currently, based on the selection of antigenically defined subsets using fluorescence-activated cell sorting (FACS), the identification of selected subpopulation cells prone to tenogenic lineage differentiation is being performed [14]. Stage-specific embryonic antigen 4 (SSEA-4) is a specific marker for pluripotent stem cells, which was first used to delineate changes during mouse embryonic development [15], and a subset of cells within the mouse adipose tissue expressing a SSEA-4 marker could be more likely to commit to desirable phenotypes. CD90 (Thy-1) was originally discovered as an antigen of T cells, thymocytes, neural cells, Kupffer's cells, and fibroblasts and could also be used as a marker for a variety of stem cells and for the axonal processes of mature neurons, which could be useful to identify and isolate ADSC subpopulations [16]. In addition, platelet-derived growth factor receptor alpha (PDGFRA) is thought to be essential for the derivation and maintenance of chondrocyte progenitor formation [17], which plays a crucial role in early embryonic mesenchymal stem cells and induces new tenocyte production for tendon regeneration and collagen organization [18]. To date, viable and homogenous subpopulation cells from human adipose tissue with superior differentiation have been successfully separated by the immunomagnetic sorting method [19, 20], serving as good candidates for biological augmentation of tissue repair. Therefore, it is valuable to explore the specific role and function of the tenogenic differentiation potential of mouse adipose-derived subpopulation cells, with positive markers SSEA-4+, CD90+, and PDGFRA+ and negative markers for hematopoietic antigens CD45-, Ter119-, CD31-, and 6C3-, on the repaired enthesis of the rotator cuff.

In this study, we aimed to identify a tenogenically differentiated subpopulation of stem cells from mouse adipose tissue for the first time. We then identified and evaluated the differentiation potential of the subpopulation cells. Furthermore, we evaluated the efficacy of adipose-derived subpopulation cells on tendon enthesis of the rotator cuff using a fibrin sealant carrier in a murine model.

## 2. Materials and Methods

### 2.1. Ethics Statement

The local animal ethics committee approved the experimental protocol for the use of mice in this study. All mice were housed under controlled conditions with free access to a normal chow diet and water. The number of mice required to evaluate rotator cuff repair was determined using a power analysis. All methods were performed according to relevant guidelines and regulations.

### 2.2. Study Design

First, three 12-week-old male pathogen-free wild-type C57Bl/6 mice (weight 24-26 g) were used for adipose tissue harvest to obtain ADSCs and the selected subpopulation cells. In addition, the subpopulation cells or ADSCs at passages 3-5 were used for in vitro differentiation, qPCR, immunofluorescence, picrosirius, and in vivo experiments. For the main study, a total of 56 animals were purchased and underwent acute unilateral detachment and transosseous repair of the supraspinatus (SS) tendon of the rotator cuff. After ADSCs and the selected subpopulation cells were collected and the fibrin sealant carrier was prepared, all mice were randomly assigned to one of the four groups with 14 mice per group: the control group without any treatment, fibrin sealant (FS) group with a fibrin carrier alone, ADSC group with 10^5^ ADSCs in a fibrin sealant carrier at the tendon-bone repair site, and the subpopulation group with 10^5^ subpopulation cells loaded with a fibrin sealant. Mice were humanely killed at four weeks to obtain supraspinatus tendon humeral complex specimens, followed by histological (*n* = 6 per group) and biomechanical analysis (*n* = 8 per group). The sample size from each group was determined before the in vivo experiments based on our power analysis and published studies in the murine rotator cuff injury model [21–23].

### 2.3. ADSC Harvest and Culture

The murine ADSCs were isolated from the subcutaneous adipose tissue in the abdomen of C57 mice according to the previous protocols [24]. In brief, subcutaneous fat from mice was dissected and carefully cleaned from adherent soft tissues. Then, fresh adipose tissue was washed with PBS solution, after which it was digested with 0.1% collagenase I solution to form chyle. Subsequently, the solution was added to complete medium at a volume ratio of 1 : 1 to stop digestion and then filtered with a 70 *μ*m cell strainer (BD Falcon, USA). After centrifugation at 300 × *g* for 10 min, the cells were suspended in a complete medium containing 10% fetal bovine serum (FBS; Gibco), 1% glutamine (Thermo, USA), and 1% penicillin/streptomycin (HyClone) and seeded into 25 cm^2^ culture flasks and cultured at 37°C in a humidified atmosphere containing 95% air and 5% CO_2_. After reaching 70% to 80% confluence, the isolated cells were trypsinized, resuspended, and passaged.

### 2.4. Adipose-Derived Subpopulation Cell Sorting and Culture

**Figure 1:**
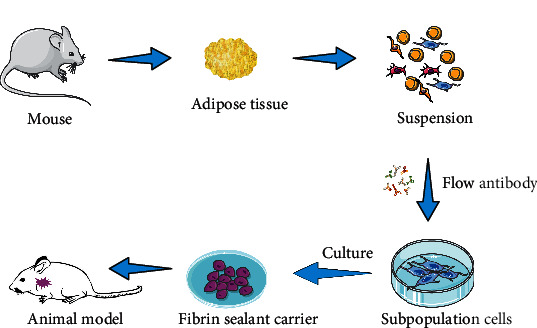
Schematic diagram showing the study design of adipose-derived subpopulation cells.

The sorting and culture conditions of the subpopulation cells are depicted in Figure 1. Briefly, after the adipose tissue was digested into chyle and centrifuged at 4°C for 5 min at 1500 rpm, the supernatant was discarded, and the remaining precipitates were washed with FACS buffer. The related surface markers of the subpopulation cells were analyzed by flow cytometry analysis as follows: the precipitates (1 × 10^6^ cells) were suspended in 100 *μ*L phosphate-buffered saline (PBS) containing 10 *μ*g/mL antibodies including Alexa Fluor® 700-conjugated CD31 (102444, BioLegend), Alexa Fluor® 700-conjugated CD45 (103128, BioLegend), Alexa Fluor® 700-conjugated Ter119 (116220, BioLegend), PerCP/Cyanine5.5-conjugated 6C3 (108316, BioLegend), PE-conjugated CD90 (555821, Biosciences, USA), PE/Cy7-conjugated PDGFRA (25-1401-80, eBioscience), and Alexa Fluor® 488-conjugated SSEA4 (330412, BioLegend). As an isotype control, nonspecific anti-mouse IgG coupled with Alexa Fluor® 700, PE, PE/Cy7, PerCP/Cyanine5.5, or Alexa Fluor® 700 (Becton Dickinson) was substituted for the primary antibody. After incubation for 30 min at 4°C, the cells were washed with PBS and resuspended in 500 *μ*L of PBS for analysis. The subpopulation cells were sorted by flow cytometry using a Beckman Coulter XL System (Beckman, USA) and analyzed with FlowJo 10 software (Tree Star, USA).

The subpopulation cells were cultured in 3 mL primary medium containing 1% antibiotic-antimycotic (Gibco), DMEM/low glucose (Gibco), and 10% fetal bovine serum (FBS; Gibco). The cells were cultured in wells (1 mL/well) on a 12-well plate. The entire medium was replaced with medium every three days. The cells were subcultured when they reached 80%–90% confluence and used for further studies.

### 2.5. Colony-Forming Unit (CFU) Assay of Subpopulation Cells

In order to determine the optimal isolation cell seeding density of the subpopulation cells, nucleated cells were cultured in 6-well plates at 50, 500, and 5000 cells/cm^2^ in the experiment; this procedure was repeated three times. On the 10th day of culture, after fixation with 4% paraformaldehyde, the cells were stained with 1% crystal violet (Solarbio, CHN) to quantify the colony formation. Distinguishable colonies larger than 2 mm in diameter were counted. Based on the maximum number of colonies obtained without contact inhibition, the optimal cell inoculation density was determined. The percentage of subpopulations was calculated by dividing the number of colonies under the optimum seeding density by the number of nucleated cells.

### 2.6. Cell Proliferation and Multidifferentiation Potential

The third passage subpopulation cells and ADSCs were collected; after counting and recording cells per mL, the cells were diluted to 75,000 per mL. Then, 100 *μ*L of cells (7500 cells in total) and 100 *μ*L medium were added to 5 wells and incubated overnight. At 1, 3, 5, 7, and 9 d, the cell counting kit-8 (CCK-8, Osaka, Japan) was used to assess the cell proliferation of the sample. The absorbance at 450 nm was measured using a microplate reader (Thermo Scientific).

The osteogenic, chondrogenic, and adipogenic differentiation potentials of the isolated cells were determined using Alizarin Red S staining (at day 14), Oil Red O staining (at day 21), and Alcian Blue staining (at day 28), respectively. In brief, cells were plated in 6-well plates at 5000 cells/cm^2^ at 37°C and 5% CO_2_ with complete medium. When the cell fusion reached 60%–70%, the osteogenic groups were treated with osteogenic differentiation medium (Cyagen, CHN), the adipogenic groups were induced with adipogenic differentiation medium (Cyagen, CHN), and the chondrogenic groups were induced with chondrogenic differentiation medium (Cyagen, CHN). At the end of differentiation, 1× PBS was used to wash the wells two times. After adding 2 mL 4% paraformaldehyde to each well to fix the contents for 30 min and washing with PBS, 1 mL Alizarin Red S, Oil Red O, and Alcian Blue solution were added to each well for 5 min. Images of stained cells were obtained using a fluorescence microscope and a light microscope.

### 2.7. Tenogenic Differentiation Test

Connective tissue growth factor (CTGF) is a growth factor that promotes the tenogenesis of cultured stem cells [25]. When the subpopulation cell or ADSC fusion reached 80%, the medium containing 25 ng/mL CTGF was refreshed in each well. The medium was replaced with fresh medium every three days.

After a 14-day culture, the expression of tenogenic lineage-specific genes (early growth response 1 (EGR1), scleraxis (SCX), and tenomodulin (TNMD)) was tested using qRT-PCR with appropriate primers (Table 1). Specifically, RNA was extracted using a RNeasy Kit (Qiagen, Hilden, Germany) according to the manufacturer's instructions. Complementary DNA was synthesized using a reverse transcriptase kit (Transgen, AT341, China), and quantitative PCR was performed using a qPCR kit (Promega, Wisconsin) and amplified on an ABI PRISM 7900HT System (Applied Biosystems, Foster City). qRT-PCR conditions for each primer were performed as follows. First, total RNA (1000 ng) was reversely transcribed into first-strand cDNA in a 20 *μ*L reaction with random primers using the TransScript All-in-One First-Strand cDNA Synthesis SuperMix Kit according to the manufacturer's instructions. PCR of 1 *μ*L of diluted cDNA from each sample was carried out in 20 *μ*L reactions containing Platinum SYBR Green qPCR SuperMix-UDG and appropriate primers in the ABI StepOne Plus System from Applied Biosystems. The PCR thermal cycle was set as follows: 10 min at 95°C for one cycle and 15 s at 95°C and 35 s at 60°C for 40 cycles. A melt curve analysis was performed at the end of each SYBR Green PCR. The efficiencies of all the primers were all over 90%. The relative gene expression was calculated using the 2 − ΔΔCT method and normalized to the levels of glyceraldehyde 3-phosphate dehydrogenase (GAPDH). Furthermore, immunofluorescence was used to assess the tenogenic differentiation. The cells were blocked with 1% bovine serum albumin and incubated with the primary antibody against tenomodulin antibody (TNMD, 1 : 200, Invitrogen, USA) at 4°C overnight. The corresponding secondary antibodies (goat anti-rabbit IgG H&L Alexa Fluor® 488, Abcam, USA) were then combined for 30 min before DAPI (H-1200, Vector Laboratories, Inc., CA, USA) was stained in the dark. The positive staining areas of the immunofluorescence of TNMD were measured by defining a region of interest (ROI) using ×10 magnification photomicrographs. Then, the positive area of the ROI was calculated with Image-Pro Plus software (version 6.0.0; Media Cybernetics Inc.).

In addition, the picrosirius red staining kit (Solarbio, CHN) was used to stain the cells to assess tenogenicity after a 21-day culture. After tenogenic differentiation was complete, 1× PBS was used to wash the wells two times. The cells in each well were fixed for 30 min with 2 mL 4% paraformaldehyde and then stained with 0.1% picrosirius red solution for 1 h at room temperature. After the staining solution was removed, the cells were thoroughly rinsed with 0.5% acetic acid. Images of stained cells were observed using a fluorescence microscope and a light microscope.

### 2.8. Animal Model

A mouse model of supraspinatus tendon enthesis healing was performed using the previously published techniques [23, 26]. Briefly, after anesthetization with an intraperitoneal injection of 0.3% pentobarbital sodium (0.6 mL/20 g; Sigma-Aldrich, St. Louis, MO), mice were placed in a right lateral decubitus position, incised, and bluntly separated to expose the deltoid under sterile conditions. After the deltoid was released with sharp dissecting scissors, the humerus was grasped to expose the enthesis of the SS tendon. Subsequently, the SS was transected with a microscalpel, and a 6-0 PDS suture (Ethicon) was placed through the SS tendon in a figure-of-eight fashion. Next, the cartilage layer in the footprint on the greater tuberosity was completely removed with a no. 11 blade to expose the spongy bone. With the humerus stabilized, the suture was used to create a tunnel within the bone of the humeral head transversely from posterior to anterior, and the entrance of the bone tunnel was made from the supraspinatus footprint on the humeral head. For the control group, the suture was directly ligated to bring the tendon back down to its original insertion site. For the other groups, fibrinogen solution (Sigma-Aldrich) and thrombin solution (Sigma-Aldrich) were prepared and then mixed with normal saline (FS group), 10^5^ subpopulation cells (subpopulation group), or ADSCs (ADSC group). The tendon was approximated by tying sutures over the original footprint. The wounds were closed using Ethicon sterile sutures. Buprenorphine (0.05 mg/kg) was administered with a subcutaneous injection for analgesic treatment, and penicillin G was administered for antibiotic treatment once a day for three days after surgery (0.05 mg/kg, subcutaneous injection).

### 2.9. Histological Experiments

Four weeks after the repair, the tendon enthesis specimens designated for histological analyses were fixed in 4% neutral buffered formalin for 24 h, decalcified in 10% ethylenediaminetetraacetate for two to three weeks, embedded in paraffin, and then sectioned at a 5 *μ*m thickness. The slices were subsequently stained with hematoxylin and eosin (H&E) and toluidine blue-fast green (TB) to detect the healing characteristics of tendon enthesis. The stained sections were analyzed by two independent blinded observers using the previously established criteria of a modified tendon-to-bone maturing scoring system to evaluate the cellularity, vascularity, continuity, cell alignment of fibrocartilage, and tidemark from 0 to 4 points, respectively [27]. The difference between each group was compared using the total score of outcomes from the 10-fold magnification histology images. A higher score indicated better bone-tendon interface (BTI) healing, and a perfect score of 20 indicated healthy attachment with excellent maturation.

### 2.10. Biomechanical Testing

Biomechanical outcomes, such as failure load (N) and ultimate stress (MPa), were used as the ultimate indices to assess the healing quality of the bone-tendon interface, which was measured using a mechanical testing machine (MTS Systems Corp., USA). Before the test, the cross-sectional area (CSA) at the insertion of the SS tendon was measured and calculated using a caliper under a tension of 0.1 N. Subsequently, the tendon was gripped using a clamp with sandpaper, while the humerus was realigned in the bottom fixture. The specimens were loaded to failure at a rate of 0.03 mm/min after a preload of 0.1 N. Failure load (N) was obtained from the recorded load-displacement curve, and the ultimate stress was calculated as the maximum load that the sample could withstand divided by the CSA. During testing, 0.9% saline was applied to prevent tissue dehydration.

### 2.11. Statistical Analysis

All quantitative data are presented as mean ± standard deviation (SD). A two-tailed unpaired Student's *t*-test was used to compare gene expression. Data on cell proliferation and biomechanical results were analyzed statistically using a one-way analysis of variance (ANOVA) with Tukey's post hoc test, while the histological scores among the groups were determined using the Mann-Whitney test. Statistical significance was set at *P* < 0.05. All data analyses were performed using SPSS software (version 17.0).

## 3. Results

### 3.1. Characteristics of the Mouse Adipose Tissue-Derived Subpopulation Cells

**Figure 2:**
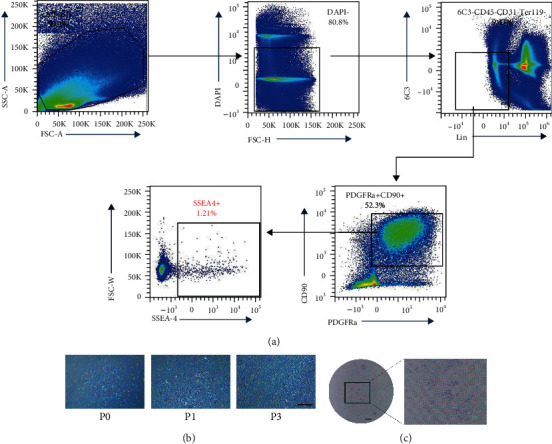
The characteristics of the subpopulation cells isolated from mouse adipose tissue. (a) Flow cytometry for the subpopulation cells. (b) Morphology of the subpopulation cells at different passages; scale bar = 200 *μ*m. (c) Colony-forming unit assay of subpopulation cells after 10 days of culture; scale bar = 2 mm.

A specific subset of cells (>1%) was successfully isolated from mouse adipose tissue with positive markers SSEA-4, CD90, and PDGFRA but negative markers for hematopoietic antigens Ter119, CD31, 6C3, and CD45 (Figure 2(a)). These immunophenotypic profiles were in accordance with the criteria for defining MSCs proposed by the International Society for Cellular Therapy (ISCT) [28]. After the primary culture, adherent cells and spindle-shaped or rounder-shaped colonies were observed (Figure 2(b)). Specifically, most of the isolated cells exhibited spindle-shaped and fibroblast-like morphology; few of them still displayed a rounder shape at passage 1. With the passage of cells, the subpopulation cells exhibited a homogeneous spindle-shaped morphology. After 10 days of culture, the subpopulation cells at passage 4 formed adherent cell colonies (7.13 ± 1.22, Figure 2(c)).

### 3.2. Proliferation and Trilineage Differentiation Potential between Subpopulation Cells and ADSCs

**Figure 3:**
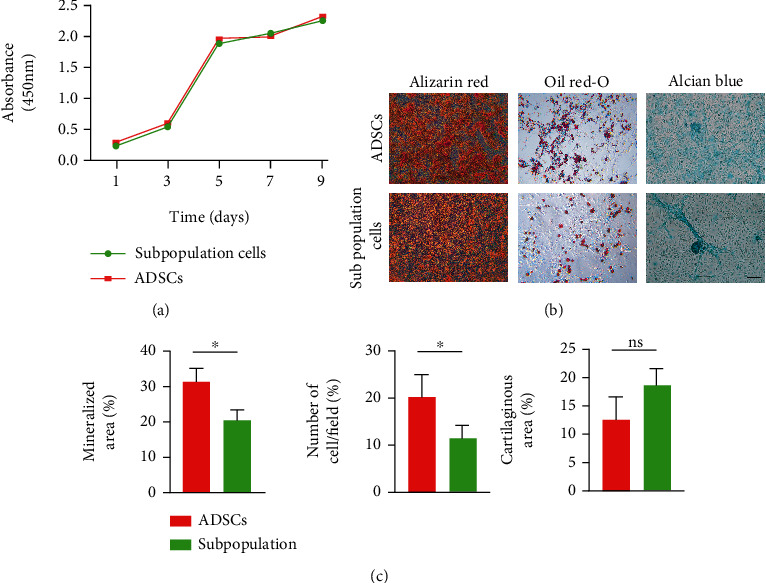
Proliferation and trilineage differentiation potential between ADSCs and the subpopulation cells. (a) Proliferation for both groups was determined using a CCK-8 assay. (b) Alizarin Red staining, Oil Red O staining, and Alcian Blue staining of the two groups after a 21-day culture. (c) Quantification of the extent of in vitro osteogenic, adipogenic, and chondrogenic differentiation between ADSCs and the subpopulation cells. Scale bar = 20 *μ*m, ^∗^*P* < 0.05. ns means no difference.

CCK-8 analysis was used to measure cell proliferation. Our results indicated that passage 3 of the isolated subpopulations proliferated at a high rate within the first nine days, and no significant differences were observed between the subpopulations of cells and ADSCs (Figure 3(a)).

For trilineage differentiation potential, the osteogenic, chondrogenic, and adipogenic differentiation of subpopulation cells was identified using positive staining for Alizarin Red, Oil Red O, and Alcian Blue. Compared to ADSCs, the subpopulation cells showed lower osteogenic and adipogenic differential potential, while no significant difference was noted in chondrogenic differential potential (Figures 3(b) and 3(c)).

### 3.3. Tenogenic Differentiation Potential

**Figure 4:**
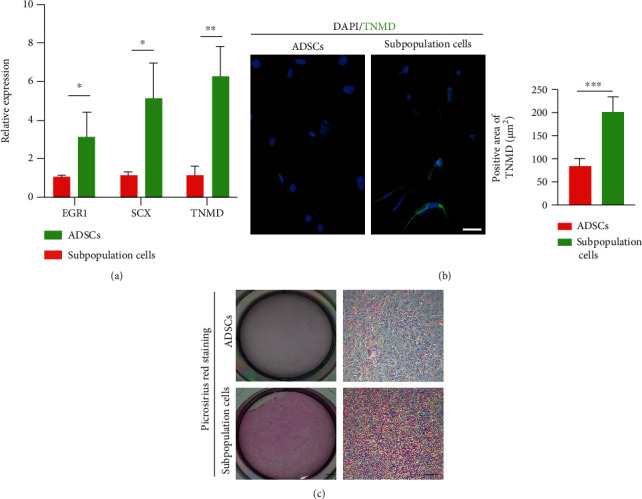
Tenogenic differentiation of ADSCs and subpopulation cells in vitro. (a) Tenogenic gene (EGR1, SCX, and TNMD) expression compared between ADSCs and the subpopulation cells. ^∗^*P* < 0.05, ^∗∗^*P* < 0.01. EGR1: early growth response1; Scx: scleraxis; Tnmd: tenomodulin. (b) Immunofluorescence for Tnmd expression of the two groups after 14 days of tenogenic induction and positive staining area of TNMD. Scale bar = 20 *μ*m. (c) Picrosirius red staining for both groups. Scale bar on the left = 1 mm, scale bar on the right = 100 *μ*m.

The isolated subpopulation cells exposed to tenogenic-induced medium for 14 days showed a significant enhancement in tenogenic gene (EGR1, SCX, and TNMD) expression compared to the ADSC group (*P* < 0.05 for all, Figure 4(a)). Specifically, the gene expression of EGR1, SCX, and TNMD increased by factors of 3.13 ± 1.26, 5.15 ± 1.81, and 6.25 ± 1.58, respectively. The immunofluorescence assay revealed that the subpopulation cells had a significantly increased expression of Tnmd, compared to that in the ADSC group (Figure 4(b)). In addition, the subpopulation cells synthesized more collagen than the ADSCs, as shown by picrosirius red staining (Figure 4(c)). The results revealed that the subpopulation cells have superior tenogenic differentiation.

### 3.4. Histological Evaluation

**Figure 5:**
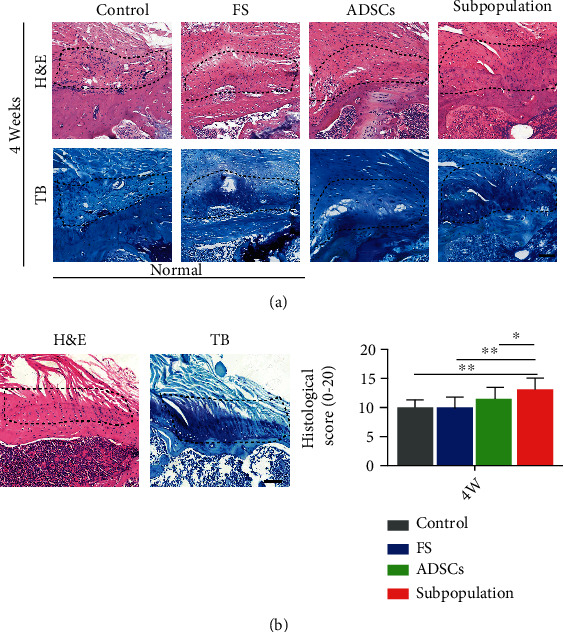
Representative pictures of the supraspinatus tendon humeral head insertion at postoperative four weeks. (a) H&E staining and TB staining images for all groups. (b) Modified tendon maturing score of the repaired enthesis. Area outlined by the dotted line represents the tendon enthesis. Scale bar = 200 *μ*m. ^∗^*P* < 0.05.

The regenerated enthesis in the rotator cuff for all groups was evaluated using H&E and TB staining. At four weeks after the operation, the interface in the control and FS groups with areas of direct tendon-bone contact through a fibrovascular scar was poorly organized and showed a few inflammatory cells, while the repaired insertion site in the ADSCs or subpopulation group contained more newborn collagen organization and became more organized. In the semiquantitative histologic scoring, based on a blinded analysis of the repaired site, no significant differences were observed between the control and FS groups. Meanwhile, the histological score in the ADSC group was slightly higher than that of the FS or control group, without a statistical difference. Unexpectedly, the subpopulation group score was significantly higher than those of the other groups (Figure 5, *P* < 0.05). The results of histological assessments showed that the subpopulation cells may promote tendon enthesis healing, as evidenced by superior bone-tendon insertion maturation in the subpopulation group.

### 3.5. Tensile Properties

In biomechanical testing (Supplementary Figure S1), all specimens ruptured at the insertion site. At four weeks after the operation, the failure load and ultimate stress in the subpopulation group were significantly higher than those in the other groups (*P* < 0.05). The failure load in the group was significantly greater than that in the control and FS groups (*P* < 0.05), while there was no significant difference in the ultimate stress. No differences in failure load and ultimate stress were found between the FS and control groups at four weeks postoperatively. Meanwhile, there was no significant difference in CSA between the groups. These outcomes indicated that the subpopulation cells were able to promote the biomechanical healing quality of tendon enthesis of the rotator cuff.

## 4. Discussion

RCT is a widespread problem in orthopedics and sports medicine that seriously affects quality of life. Clinically, arthroscopic surgery is usually performed to reconstruct the tendon to the humeral footprint. Due to the high retear rate, mesenchymal stem cells have attracted considerable attention as regenerative medicinal agents for rotator cuff healing [29]. For example, ADSCs are easily obtained from the rich fat tissue of several animals, including humans, mice, rats, canines, and cattle, and have the capacity to differentiate into tendon fibroblasts, bone, and cartilage to augment the healing response [30–33]. However, the high intrinsic heterogeneity of ASCs hinders the translation of MSC-based therapies into the clinic [14]. To date, stem cell therapies for RCT have not yielded satisfactory results, thus necessitating the development of a potential solution to the problem of tendon enthesis healing in the rotator cuff.

It is necessary to select a suitable cell type for transplantation, which should ideally possess high proliferative capacity and tissue specificity. Recently, more attention has been paid to the selection of antigenically defined subpopulations of stem cells sorted based on the expression of specific surface molecules [34, 35]. Previous studies have shown that CD271+ MSCs from the synovial membrane demonstrated excellent cartilage repair, while CD105-depleted ADSCs showed great osteogenic differentiation [16]. In the present study, SSEA-4+CD90+PDGFRA+ subpopulation cells were successfully isolated directly from the adipose tissue fractions, avoiding the processes of early marker changes associated with plate culture. Meanwhile, better characterization and standardization of the subpopulation cells could assure the safety of cell therapies and speed up the progress of the field from bench to bedside. On the other hand, the previous studies have shown that SCX is critically involved in the development of tendon progenitors, and EGR1 is an essential transcription factor for the subsequent tendon collagen fiber differentiation and maturation [36]. The gene expression of TNMD is widely regarded as a late-stage tenogenic marker [37, 38]. The subpopulation cells showed promise for enhancing tenogenic differentiation with respect to the unsorted ADSCs, as evidenced by the significant upregulation of SCX, TNMD, and EGR1 expression. Interestingly, collagen deposition was also elevated in the sorted SSEA-4+CD90+PDGFRA+ subpopulation cells, as shown by picrosirius red staining. These findings may suggest that the subpopulation cells extracted from adipose tissue can be an ideal cell source for tendon or ligament repair in vivo.

A fibrin sealant, well known as a useful delivery carrier matrix, has been widely applied in various traumas and regenerative medicine fields. Furthermore, previous studies indicated that using a fibrin sealant as a degradable graft scaffold could effectively slow the release of biologically active factors and avoid local explosive release [39, 40]. Gulotta et al. reported the addition of MSCs loaded with a fibrin sealant to the healing rotator cuff insertion site to determine the effect of cell-based strategies. In our study, we used a method to load ADSCs and sorted subpopulation cells via the absorbable fibrin sealant with a custom size and shape, demonstrating their therapeutic efficacy in a murine rotator cuff injury model. In addition, a control group without any treatment and an FS group without any MSCs were designated to eliminate the effect of the fibrin sealant on rotator cuff healing, and no positive or negative effects of the fibrin sealant on the rotator cuff healing process were identified histologically or biomechanically.

Our study had several limitations. First, although rodents are frequently utilized as a model of acute rotator cuff tears, this method does not reflect the clinical processes of chronic degeneration tendon rupture, which can result in muscle atrophy, fibrosis, fatty degeneration, and tendon retraction occurring in human patients. Second, we could not determine the specific growth factors or cytokines secreted from ADSCs or subpopulation cells that promoted rotator cuff healing in vivo, and future studies may be needed to explore the special role of MSC-related growth factors or cytokines in the healing site. Third, we evaluated tendon enthesis regeneration at a single time point. It is possible that implantation of the subpopulation cells could have demonstrated a more profound effect on RC healing over a longer period. Finally, we investigated only a single dose of ADSCs or subpopulation cells preloaded with the fibrin sealant for implantation; more animals and optimized doses would result in better results and decreased error bars.

## 5. Conclusion

In conclusion, for the first time, SSEA-4+CD90+PDGFRA+ subpopulation cells were successfully isolated from the adipose tissue fractions directly, showing clonogenicity and high proliferation capacity. In addition, these subpopulation cells express specific markers of MSCs and can differentiate into tenogenic potential. Furthermore, we investigated the effects of subpopulation cells on the healing process of tendon enthesis in a mouse rotator cuff model. The subpopulation of cells could enhance the early stage of repaired tendon enthesis with improved collagen fiber organization and biomechanical strength.

## Figures and Tables

**Figure 6:**
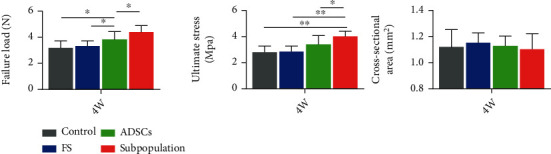
Biomechanical properties of the repaired enthesis in the rotator cuff, including failure load, ultimate stress, and CSA for all groups at postoperative week 4. CSA: cross-sectional area. ^∗^*P* < 0.05.

**Table 1:** Primer sequences used for qRT-PCR analysis.

Markers of tenogenic genes	Primer sequence (5′-3′)
EGR1
	Forward TCGGCTCCTTTCCTCACTCA
	Reverse CTCATAGGGTTGTTCGCTCGG
SCX	Forward CTGGCCTCCAGCTACATTTCT Reverse GTCACGGTCTTTGCTCAACTT
TNMD	Forward ACACTTCTGGCCCGAGGTAT Reverse GACTTCCAATGTTTCATCAGTGC
GAPDH	Forward AGGTCGGTGTGAACGGATTTG Reverse TGTAGACCATGTAGTTGAGGTCA

## Data Availability

The data used to support the findings of this study are included in the article.

## Supplementary Materials

Supplementary Materials Supplementary Figure S1: biomechanical test of the regenerated rotator cuff in mice. (A) Gross picture of the supraspinatus tendon enthesis for the biomechanical test and (B) an example of the load-displacement curve.
