# Supplementary material for: Enhanced Repaired Enthesis Using Tenogenically Differentiated Adipose-Derived Stem Cells in a Murine Rotator Cuff Injury Model
Source: Stem Cells Int. 2022 May 14;2022:1309684. doi: 10.1155/2022/1309684 (PMC9124132; doi:10.1155/2022/1309684)
Supplement: Supplementary Materials — Supplementary Figure S1: biomechanical test of the regenerated rotator cuff in mice. (A) Gross picture of the supraspinatus tendon enthesis for the biomechanical test and (B) an example of the load-displacement curve. [file 1309684.f1.docx]

**Supplementary Material**

| 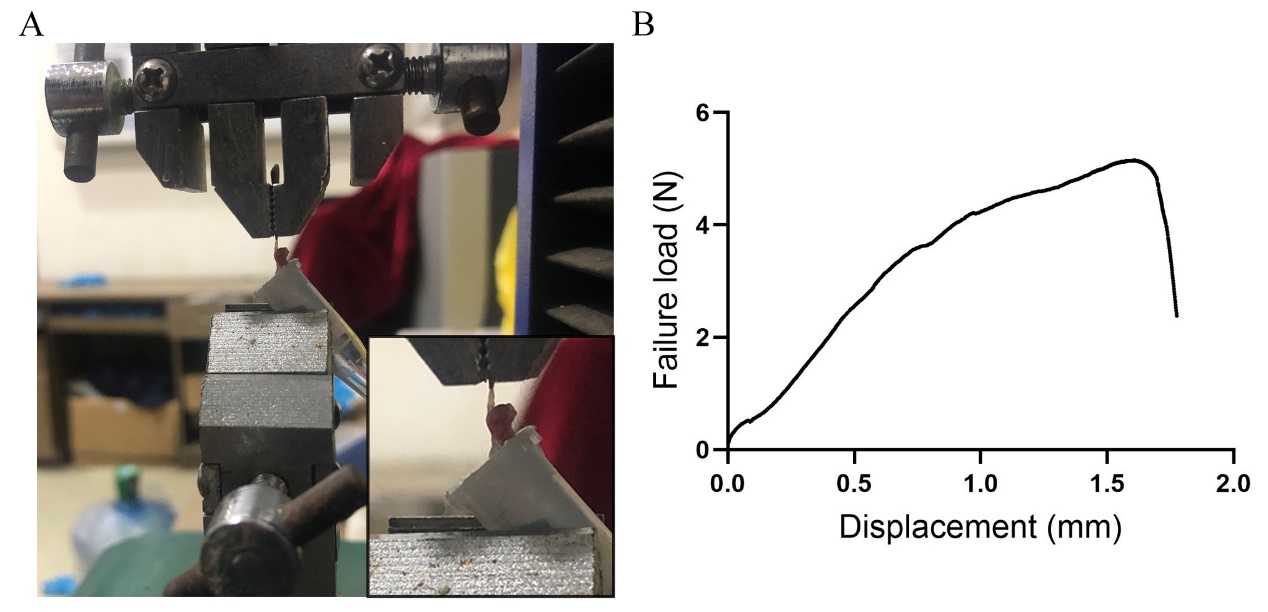 |
| --- |
| **Figure S1** Biomechanical test of the regenerated rotator cuff in mice. A) gross picture of the supraspinatus tendon enthesis for the biomechanical test, and B) an example of the load-displacement curve. |
